# Lysine 63-Polyubiquitination Guards against Translesion Synthesis–Induced Mutations

**DOI:** 10.1371/journal.pgen.0020116

**Published:** 2006-07-21

**Authors:** Roland K Chiu, Jan Brun, Chantal Ramaekers, Jan Theys, Lin Weng, Philippe Lambin, Douglas A Gray, Bradly G Wouters

**Affiliations:** 1 Department of Radiation Oncology, GROW Research Institute, University of Maastricht, Maastricht, Netherlands; 2 Ottawa Health Research Institute, Ottawa, Ontario, Canada; Brandeis University, United States of America

## Abstract

Eukaryotic cells possess several mechanisms to protect the integrity of their DNA against damage. These include cell-cycle checkpoints, DNA-repair pathways, and also a distinct DNA damage–tolerance system that allows recovery of replication forks blocked at sites of DNA damage. In both humans and yeast, lesion bypass and restart of DNA synthesis can occur through an error-prone pathway activated following mono-ubiquitination of proliferating cell nuclear antigen (PCNA), a protein found at sites of replication, and recruitment of specialized translesion synthesis polymerases. In yeast, there is evidence for a second, error-free, pathway that requires modification of PCNA with non-proteolytic lysine 63-linked polyubiquitin (K63-polyUb) chains. Here we demonstrate that formation of K63-polyUb chains protects human cells against translesion synthesis–induced mutations by promoting recovery of blocked replication forks through an alternative error-free mechanism. Furthermore, we show that polyubiquitination of PCNA occurs in UV-irradiated human cells. Our findings indicate that K63-polyubiquitination guards against environmental carcinogenesis and contributes to genomic stability.

## Synopsis

Genome instability is associated with increased cancer risk, and thus considerable effort has been put into unraveling the mechanisms underlying genome surveillance. Guarding the integrity of DNA are a number of DNA-repair and cell cycle–control systems. Insight into how these pathways become activated is crucially important to the understanding of carcinogenesis and in the development of cancer treatments. This study concerns a distinct pathway that promotes the tolerance of DNA damage during its replication phase. Prior attempts to investigate this pathway in human cells have been difficult due to extensive redundancy in the genes that carry out this process. Previous knowledge from lower organisms suggested the requirement for enzymes capable of constructing a chain of ubiquitin molecules linked in a specific manner. The authors used a novel approach to disrupt the formation of these ubiquitin chains in human cells and found that this caused a significant increase in mutations after exposure to UV light. Several lines of evidence implicate a family of error-prone enzymes, called translesion synthesis polymerases, in the formation of these mutations. Furthermore, they provide evidence suggesting that proliferating cell nuclear antigen (PCNA), a protein found at sites of replication, is the relevant target of these chains in human cells. These findings indicate that polyubiquitination guards against environmental carcinogenesis and contributes to genomic stability.

## Introduction

In contrast to DNA-repair pathways, DNA damage tolerance (DDT) is characterized by bypass of DNA lesions rather than their direct removal or repair. The DDT pathway is likely responsible for the ability of cells to continue to proliferate with tremendous amounts of damage in their genomes [1]. The genetic and mechanistic basis of DDT is best understood in yeast, where it is known to be an extremely important determinant of the toxicity and mutagenicity of many DNA-damaging agents [2,3]. Often referred to as RAD6-dependent repair or post-replication repair, DDT requires interaction of the E2 ubiquitin (Ub) conjugase RAD6 and the E3 Ub ligase RAD18 at sites of DNA damage [4]. Here they mediate mono-ubiquitination of proliferating cell nuclear antigen (PCNA) at K164 and subsequent recruitment of a specialized translesion synthesis (TLS) polymerase capable of replication past the lesion [5,6]. Several yeast and mammalian TLS polymerases have been identified, including POLη (RAD30A), POLι (RAD30b), REV1, REV3, and POLκ [7]. These are highly error-prone polymerases that allow replication past a variety of DNA lesions [7]. POLη plays a uniquely important role in the repair of UV damage as it mediates error-free bypass of thymine–thymine dimers, the most common UV-induced lesion [8]. *Saccharomyces cerevisiae RAD6* and *RAD18* mutants that are unable to carry out DDT are highly sensitive to various genotoxic agents including UV irradiation and methyl methane sulfonate (MMS) [9]. These mutants also show a reduction in UV-induced mutations [10] that arises due to the inability to recruit the error-prone TLS polymerases [11].

Genetic epistasis studies in yeast have established a second arm of the DDT pathway that is distinct from TLS and is referred to as damage avoidance [5,12–14]. This pathway is also downstream of RAD6/RAD18, but in contrast to the error-prone TLS pathway resolves blocked replication forks through an error-free manner. Its mechanism is not fully understood, but may involve fork reversal and recombination with the undamaged replicated sister chromatid [5]. This damage-avoidance pathway requires a second ubiquitination complex composed of RAD5 and the UBC13/MMS2 heterodimer [5]. UBC13/MMS2 is a unique Ub conjugase that synthesizes polyUb chains linked through K63–G76 bonds rather than through the typical K48–G76 bonds [13]. Although lysine 63-linked polyubiquitin (K63-polyUb) chains can serve as competent proteolytic signals, they are less efficient at targeting substrates to the proteasome than K48-linked chains [15], and the proteolytic activity of the proteasome may not be required for error-free repair [13]. In yeast, a model has emerged in which error-free damage avoidance occurs when mono-ubiquitinated PCNA becomes further modified by K63-polyUb via RAD5 and MMS2/UBC13. Interestingly, modification of K164 in PCNA by sumoylation rather than by ubiquitination reduces homologous recombination [16,17].

There is convincing evidence that the DDT pathway, and particularly the TLS arm, is also important in higher eukaryotes including humans. Mouse and human homologs of *RAD6, RAD18, PCNA,* and many of the TLS polymerases have been identified [18]. The TLS polymerases form foci at sites of DNA damage following UV irradiation and are associated with other proteins in the replication machinery [19]. As in yeast, RAD6 and RAD18 mediate mono-ubiquitination of PCNA at K164 in UV-irradiated mammalian cells in a dose- and time-dependent manner [11]. Mono-ubiquitination of human PCNA has been suggested to provide a signal for polymerase switching since it leads to its increased association with POLη via its ubiquitin-binding domain (UBD) or the UBZ (ubiquitin-binding zinc-finger) in this TLS polymerase [20]. In vitro studies have also demonstrated that mono-ubiquitination of PCNA in yeast can stimulate the activities of both POLη and REV1 [21]. Recently, the deubiquitinating (DUB) enzyme USP1 was shown to directly remove the monoUb from PCNA, leading to the suggestion that USP1 is required to suppress the error-prone activity of TLS [22]. The functional importance of TLS is exemplified by the fact that mutations in *POLη* are responsible for the variant form of Xeroderma Pigmentosum (XP), a disease characterized by a 2,000-fold increased risk of developing skin cancer [8]. In contrast to other XP patients, those with the variant form (XPV) of Xeroderma Pigmentosum have no defect in excision repair [8], but are deficient in post-replication repair [23]. Furthermore, they display enhanced mutation at T–T sites, owing to usage of an alternative error-prone TLS polymerase [24].

In contrast to TLS, the importance of the damage-avoidance arm of DDT in mammalian cells is not yet firmly established. Perhaps the strongest evidence supporting a role for this pathway comes from Li et al., who showed that antisense inhibition of hMMS2 resulted in an increase in mutation frequency [25]. Nonetheless, several open questions remain to be resolved. First, a human homolog of *RAD5* has not yet been identified. This may be due to the fact that yeast RAD5 contains a helicase activity required for its function in DNA double-strand break repair, but is unimportant for DDT [26]. These authors speculated that *RAD5* in higher organisms may have evolved to lose this domain. Second, although homologs of *MMS2* exist *(hMMS2* and *hCroc1)* and are able to functionally complement loss of yeast MMS2 [27], they are additionally required for polyubiquitination of proteins in pathways unrelated to DDT [28]. Third, although evidence for human PCNA mono-ubiquitination is strong [11,29], there is less evidence for its polyubiquitination. High molecular weight bands in PCNA Western blots were noted in mouse fibroblasts following UV irradiation [11]. However, Kannouche and colleagues found no evidence for polyubiquitination in human fibroblasts [29]. They concluded that polyUb forms of PCNA were either insignificant, occurred only at low levels, or were rapidly turned over [29]. Thus, whether polyubiquitination of PCNA and subsequent activation of an error-free damage-avoidance pathway is evolutionarily conserved in humans is a source of uncertainty that we sought to resolve.

Here, we provide evidence that the ability to create K63-based polyUb chains is required for an error-free damage-avoidance pathway in human cells. We implicate this ubiquitination step in a pathway that contributes to genomic stability by suppressing translesion polymerase–mediated mutagenesis. Moreover, we show that DNA damage–induced PCNA polyubiquitination is indeed conserved in human cells, suggesting that this Ub-based molecular switch plays a decision role in directing repair in either an error-free or error-prone manner.

## Results

### Dominant Negative Approach to Disrupt K63-PolyUb Chain Assembly

In order to directly investigate the functional importance of K63-linked polyUb chains in DDT, we employed a strategy similar to that first described in yeast, to specifically inhibit assembly of these chains. In yeast, replacement of Ub with a mutant in which lysine 63 is mutated to arginine *(K63R)* disrupts the error-free arm of DDT and results in a phenotype equivalent to loss of UBC13 or MMS2 [14]. The *K63R* mutation disrupts K63-polyUb chain assembly, but has no effect on K48-linked chains that mediate proteasomal-based protein turnover [14]. In human cells, an equivalent knock-in approach is not feasible because Ub is expressed from multiple genes. The *UBA52* and *UBA80* genes encode a Ub monomer fused in frame with ribosomal subunits, while the *UBB* and *UBC* genes encode variable-length linear polymers of (typically three to four Ub and nine Ub proteins, respectively) [30]. The fusion proteins are cleaved by DUB enzymes to release individual Ub monomers.

**Figure 1:**
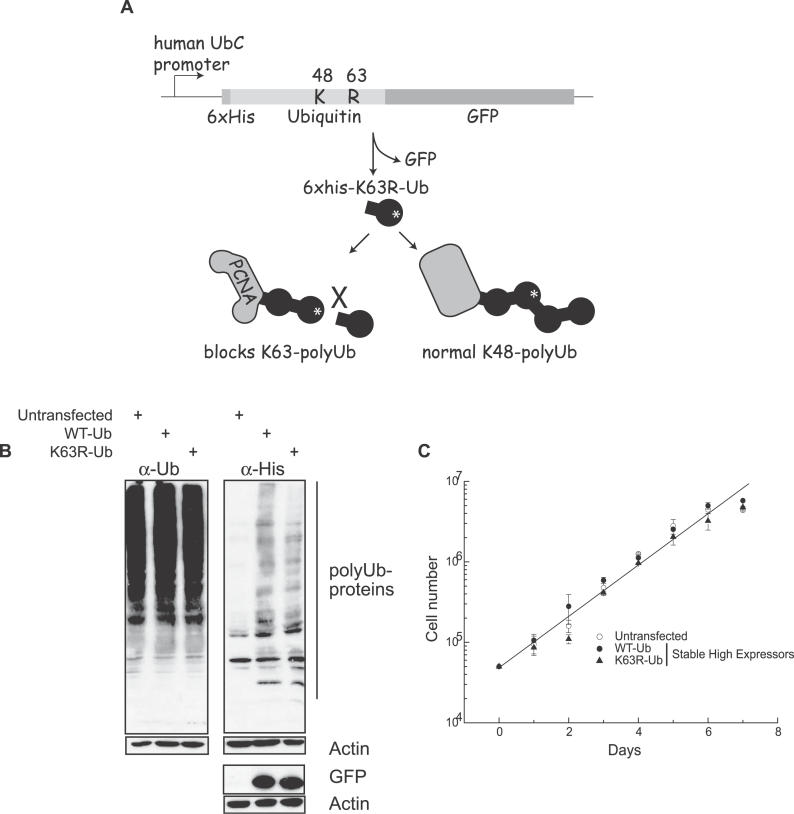
Disruption of K63-PolyUb Chain Assembly (A) Cartoon depicting dominant negative *K63R-Ub-GFP* construct. The expressed fusion protein is processed by endogenous Ub proteases generating free GFP used for detection on a flow cytometer and mono-K63R-Ub. Incorporation of this mutant will terminate K63-polyUb chains while not affecting canonical K48-polyUb chain assembly. (B) Whole-cell lysates were isolated from untransfected cells, and from cells stably expressing either *WT-Ub* or *K63R-Ub,* followed by immunoblot analysis with antibodies directed against Ub, His, and GFP. (C) The growth of untransfected, WT-Ub, or K63R-Ub cells was followed by cell counting over the course of 7 d.

Our approach was to express the *K63R-Ub* mutant in *trans* so that it competed with wild-type (WT) Ub for inclusion into polyUb chains. Its incorporation blocks further ubiquitination through K63 and thus acts in a dominant way. In a previous study, we validated and used this approach to specifically suppress K48-linked Ub chains by expressing a *K48R-Ub* mutant [31]. This same construct has also been used to inhibit K48 polyubiquitination in transgenic mice [32]. Here, we expressed a six-his-tagged *K63R-Ub* or *WT-Ub* fused in frame with *GFP* from the *UbC* promoter (Figure 1). Expression yields a fusion protein that is cleaved, releasing a six-his-tagged Ub monomer and free GFP (Figure 1B). GFP was used to sort pools of cells with stable high expression of the transgene. Both WT-Ub and K63R-Ub monomers were efficiently incorporated into polyUb chains as evidenced by their detection in high molecular weight smears characteristic of the heterogeneity of ubiquitinated proteins (Figure 1B). The *K63R-Ub* mutant did not affect normal cell proliferation as demonstrated by the identical growth rates in the sorted stable high *K63R-Ub-GFP–*expressing pools and in the similarly sorted *WT-Ub-GFP*–expressing and the untransfected cells (Figure 1C). Furthermore, disrupting K63-polyUb chain formation did not alter normal proteasome-mediated protein degradation of p53 or HIF1α (unpublished data). These data indicate that the K63R-Ub fusion protein is properly processed into K63R-Ub monomers, incorporates normally into chains, and does not alter the ability of the proteasome to recognize polyubiquitinated substrates targeted for degradation.

### Disruption of K63-PolyUb Chain Assembly Sensitizes Cells to Cisplatin—but Not UV—Induced Cell Death

**Figure 2:**
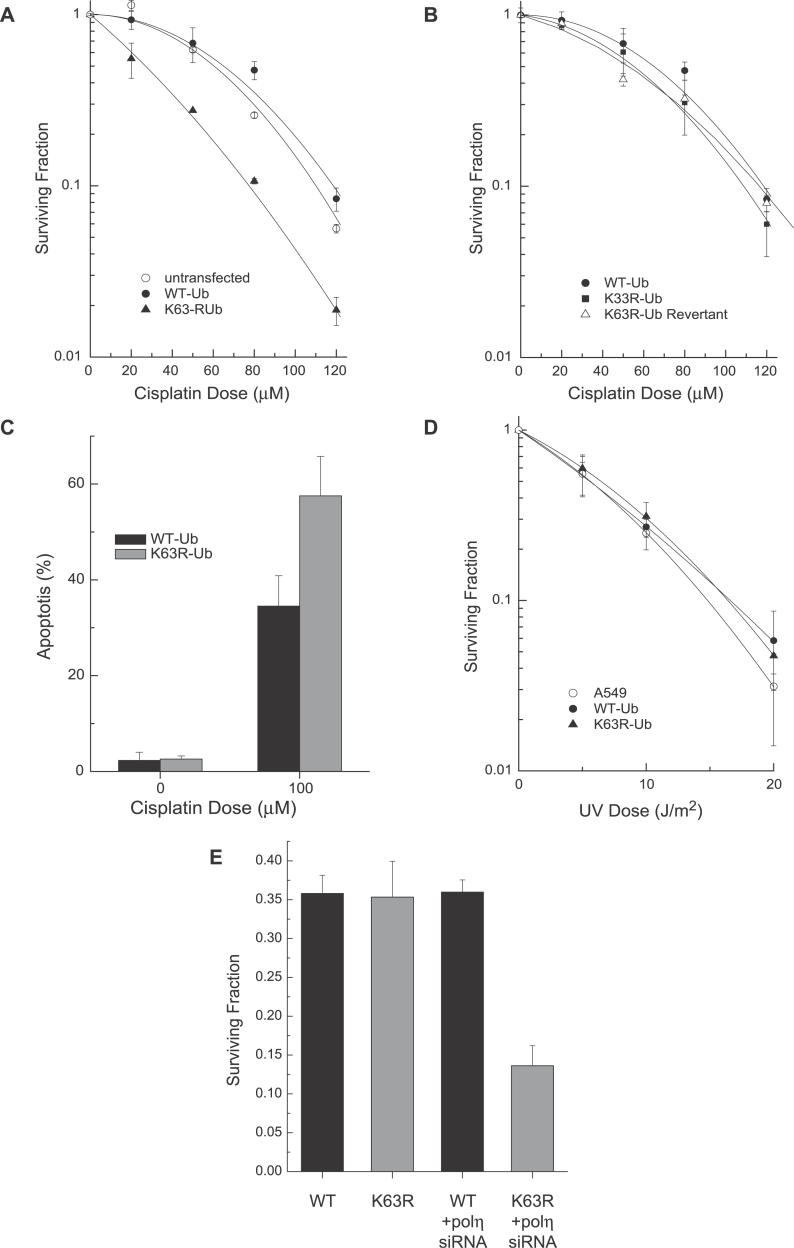
Cells Deficient in K63-Ub Chain Formation Are Sensitized to Cisplatin Treatment while UV Sensitivity Is Revealed only upon POLη Knockdown (A and B) Clonogenic survival assays were used to determine sensitivity to 1 h acute treatment with cisplatin in untransfected A549 cells or in A549 cells stably expressing *WT-Ub* or *K63R-Ub.* The mean values of three independent experiments are shown with standard error of the mean (error bars). Cells expressing *K33R-Ub* or cells that lost *K63R-Ub* expression revert to WT-Ub cisplatin sensitivity. (C) Cells were treated for 24 h with 100 μM cisplatin followed by Hoechst staining to detect apoptosis. The mean values of three independent experiments are shown with standard deviation. (D) Clonogenic survival assays were used to determine sensitivity to UV irradiation in untransfected A549 cells or in A549 cells stably expressing *WT-Ub* or *K63R-Ub.* (E) Clonogenic survival of A549 cells stably expressing *WT-Ub* or *K63R-Ub* with or without POLη RNAi following 10 J/m^2^ UV treatment.

Creation of stable cell lines expressing *WT-Ub* or *K63R-Ub* allowed us to examine the role of K63-polyUb chain assembly during recovery from DNA damage. We first investigated whether inhibition of K63-polyubiquitination would sensitize cells to agents known to sensitize yeast mutants in the error-free damage-avoidance arm of DDT [2,3]. We found that cisplatin, a chemotherapeutic agent highly toxic to yeast mutants in this pathway [2,3], is also significantly more toxic to A549 cells expressing *K63R-Ub* (Figure 2). This sensitivity is specific to expression of *K63R-Ub* since the response of cells expressing either *WT-Ub* or *K33R-Ub* is identical to that of untransfected controls (Figure 2A and 2B). This effect was not mediated by a general inhibition of ubiquitination since A549 cells expressing the *K48R-Ub* mutant are not sensitized (unpublished data). Furthermore, a K63R-Ub clone that lost expression of the transgene (as evidenced by a low GFP signal) returned to normal sensitivity (Figure 2B). These data imply that K63-polyUb chain assembly is essential for recovery from at least a subset of cisplatin-induced lesions.

We also examined the functional importance of K63-polyubiquitination in the recovery from UV-induced damage. In contrast to the data with cisplatin, the cell line with stable expression of *K63R-Ub* exhibited a dose response to UV irradiation that was identical to the parental cells or to cells expressing *WT-Ub* (Figure 2D). Thus, despite evidence that K63-polyUb chains are required for cisplatin tolerance, we found no evidence that disruption of K63-polyUb chain assembly on its own influences UV toxicity. A possible explanation for this lack of sensitivity to UV irradiation is that cells can compensate for loss of K63-polyUb–dependent repair through increased utilization of the error-prone TLS arm of the pathway. A similar situation occurs in yeast where inhibition of the error-free damage-avoidance arm of DDT results in a much milder UV sensitivity than mutations in *RAD6* or *RAD18* which additionally prevent TLS [33]. Using siRNA, we were able to knock down expression of POLη by ~13-fold (Figure S1). Similar to inhibition of K63-polyubiquitination, knockdown of POLη had no effect on UV sensitivity on its own. This observation is not unexpected since XPV cells (defective in POLη) are not sensitive to killing by UV irradiation. In contrast, knockdown of POLη in cells also expressing *K63R-Ub* did cause increased cell kill after UV treatment (Figure 2E). This increase in UV sensitivity suggests that K63-polyUb and POLη function in distinct, complementary pathways that mediate recovery from UV-induced damage.

### Disruption of K63-PolyUb Chain Assembly Increases UV-Induced Mutations

**Figure 3:**
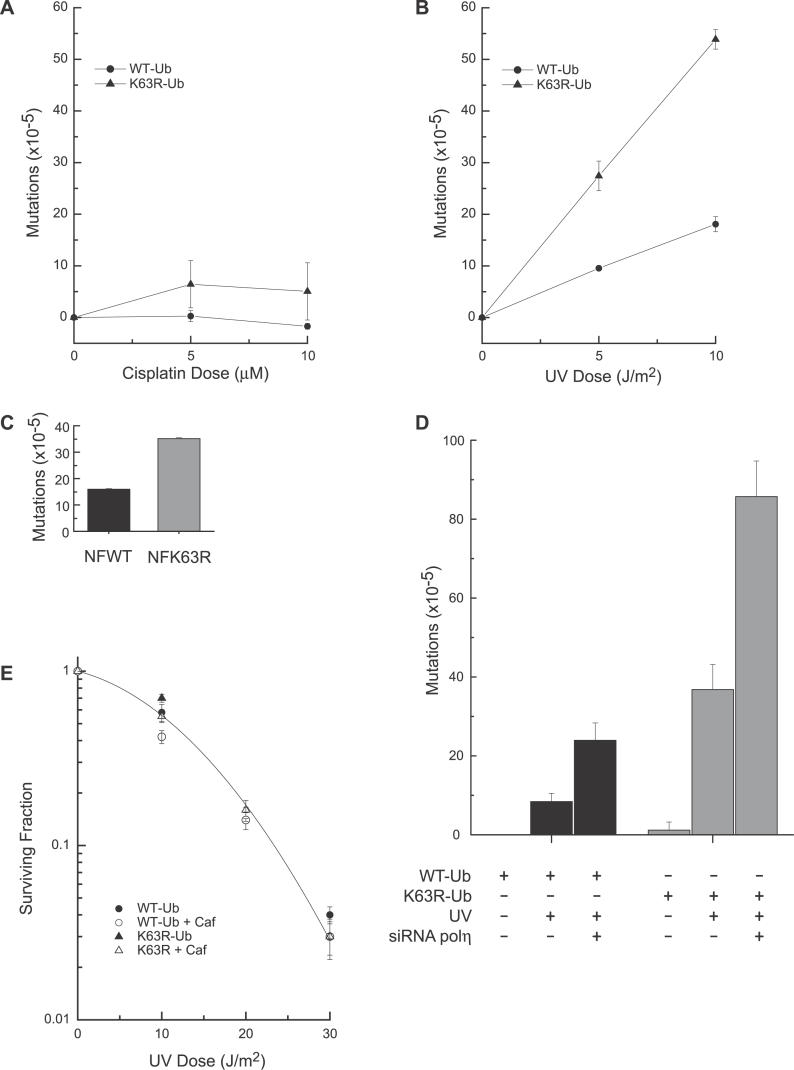
Cells Deficient in K63-Ub Chain Formation Are Mutagenic in Response to UV Treatment (A and B) Cells were treated with cisplatin for 1 h or UV irradiation and subcultured for 7 d. Cells were then plated and grown in 6-TG to select for *HPRT* mutants. The mean values of three independent experiments are shown with standard deviation. (C) Normal fibroblasts stably expressing *WT-Ub* or *K63R-Ub* were UV-irradiated (10 J/m^2^) and cultured for 5 d. Cells were then plated and grown in 6-TG to select for *HPRT* mutants. (D) The number of *HPRT* mutants was quantitated for A549 cells stably expressing *WT-Ub* or *K63R-Ub* with or without POLη RNAi. Cells were treated as described in Figure 3C. (E) Cells were UV-irradiated and plated in the absence or presence of 0.4 mM caffeine. The mean values of three independent experiments are shown with standard error of the mean (error bars).

Disruption of the error-free arm in yeast is also known to result in a dramatic increase in UV-induced mutations that is synergistic with the TLS mutant, REV3 [34]. If playing a similar role in mammalian cells, inhibition of K63-polyubiquitination should also increase UV-induced mutations. We thus analyzed mutation induction at the *HPRT* locus after UV irradiation and cisplatin exposure in these same cell lines as well as in normal human fibroblasts expressing *WT-Ub* or *K63R-Ub* (Figure 3). Consistent with this hypothesis, A549 cells expressing *K63R-Ub* show a 2.5-fold increase in UV-induced mutations compared to cells expressing *WT-Ub* (Figure 3B), and a similar increase (2.2-fold) is observed in normal fibroblasts (Figure 3C). Untransfected and *WT-Ub*–expressing cells have similar mutation frequencies (unpublished data). The increase in mutations upon inhibition of K63-polyubiquitination is consistent with a recent report that used antisense to suppress the expression of MMS2 in human cells [25]. Similar to the cells expressing *Ub-K63R,* loss of MMS2 led to an ~2-fold increase in UV-induced mutations without increasing UV-induced cell death [25]. Thus, both the enzyme that is implicated in the synthesis of K63-polyUb chains, and the chains themselves, are required for recovery from UV damage through a pathway that prevents mutations.

### Increases in UV-Induced Mutations Are Due to Increased Utilization of TLS

Many of the TLS polymerases are known to be important contributors to UV-induced mutagenesis as is illustrated by a reduction in mutation frequency when inactivated in yeast [35–38]. The data presented thus far are consistent with a model in which inhibition of K63-polyubiquitination increases UV-induced mutations owing to increased use of the error-prone branch of the TLS pathway. However, the possibility that *K63R-Ub* expression in some way increases mutations by affecting the function of one or more TLS polymerases cannot be ruled out. In fact, the phenotype of cells expressing *K63R-Ub* is similar to that described for XPV cells. Both cell types display an increase in UV-induced mutations with no significant change in UV-induced cell death. In XPV cells, this is due to loss of POLη which replicates past T–T dimers in an error-free manner [39]. Defects in POLη can be revealed by a significant increase in UV sensitivity when irradiated in the presence of caffeine, an assay used to establish the XPV phenotype [40]. However, we found that cells expressing *K63R-Ub* are not similarly hypersensitive to this combined treatment (Figure 3E), suggesting no overt defect in POLη function in these cells.

In contrast, our data suggest that POLη and K63-polyUb chains participate in separate, alternative pathways for recovery from UV-induced DNA damage. Consistent with this idea, knockdown of POLη in combination with the inhibition of K63-polyUb chain assembly resulted in both an increased toxicity to UV irradiation (Figure 2E) and in a further increase in UV-induced mutations (Figure 3D). Interestingly, the number of mutations in cells following knockdown of POLη in combination with inhibition of K63-polyUb chain assembly were far greater than additive. As expected, loss of POLη, which replicates past T–T dimers with high fidelity, resulted in a large induction in UV-induced mutations in *WT-Ub*–expressing cells (Figure 3D). These mutations are likely due to the activity of alternative TLS polymerases that can substitute for POLη, but which are error-prone across T–T dimers [41]. Additional suppression of K63-polyUb chain assembly increased the number of UV-induced mutations by 3.5-fold. This synergistic increase in mutations strongly suggests that the inability to form K63-polyUb chains places a greater requirement on the TLS pathway, and thus POLη; it is also likely that there will be a greater requirement for other lesion bypass polymerases such as POLζ [42,43] for recovery from UV damage. Moreover, the synergistic increase in mutations suggests that a significant proportion of the repair is normally carried out by the error-free component of the damage-avoidance pathway.

**Figure 4:**
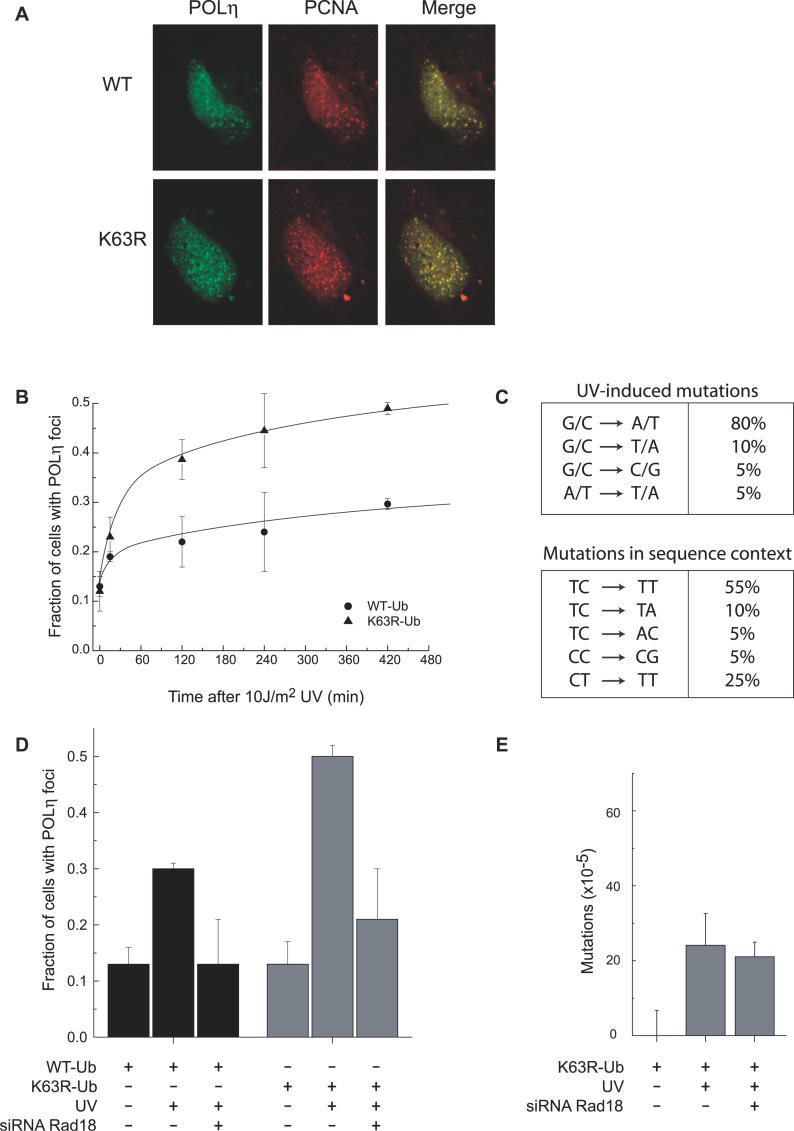
Disrupting K63-PolyUb Chain Formation Increases Reliance of Cells on the Error-Prone TLS Pathway (A) HeLa cells stably expressing *WT-Ub-puro* or *K63R-Ub-puro* were transiently transfected with a plasmid expressing a *POLη-GFP* fusion. Twenty-four hours post-transfection, cells were UV-irradiated (10 J/m^2^). POLη (green) and PCNA (red) were detected using antibodies. Shown are representative confocal photographs of cells 6 h post-UV treatment. (B) Kinetics of POLη foci formation in *WT-Ub–* and *K63R-Ub–*expressing HeLa cell lines. (C) *HPRT* mutation spectra. RNA was isolated from 6-TG resistant 10 J/m^2^ UV-treated clones followed by RT-PCR and sequence analysis of the *HPRT* locus. The UV-induced mutations are shown in the upper table. Most of the point mutations were G→A or C→T transitions indicated as G/C→A/T. The lower table in (C) shows the same mutants in sequence context. (D) Foci were quantitated 6 h post-UV treatment using a live-cell imaging fluorescent microscope. (E) The number of *HPRT* mutants was quantitated for A549 cells stably expressing *K63R-Ub* with or without RAD18 RNAi. Cells were treated as described in Figure 4B.

To further investigate the relationship between inhibition of K63R-polyUb chain assembly and TLS, we examined the spatial dynamics of the TLS polymerase POLη. This polymerase is recruited to sites of damage and can be visualized in discrete foci that co-localize with PCNA [44]. We analyzed the effects of *K63R-Ub* expression on POLη foci formation in live cells using a *POLη-GFP* fusion construct [44] (Figure 4). Since our original cells co-expressed *GFP,* we generated new stable lines from both A549 and HeLa cells expressing *WT-Ub* or *K63R-Ub* fused with the puromycin-resistance gene. These cell lines are phenotypically equivalent to the original *GFP*-expressing cells (an ~3-fold increase in *HPRT* mutants in cells expressing *K63R-Ub* compared to *WT-Ub*). Similar to previous observations [44], the majority of nonirradiated cells show homogenous nuclear distribution of the tagged polymerases (Figures 4 and S2). Foci were observed in ~11%–12% of cells and likely represent sites of ongoing replication [44]. When treated with 10 J/m^2^ UV irradiation, the percentage of cells with foci increased to 30% in cells expressing *WT-Ub* and to 49% in cells expressing *K63R-Ub* 6 h posttreatment (Figure 4B and 4D). This corresponds to an ~2-fold increase in UV-induced foci as a consequence of inhibition of K63-polyubiquitination (2.4-fold increase over background for *WT-Ub* versus 4.6-fold for *K63R-Ub, p* < 0.007).

We also analyzed the co-localization of these foci with sites of DNA replication as revealed by positive PCNA foci. We found that in both *WT-Ub* and *K63R-Ub*–expressing cells, 100% of the UV-induced POLη foci co-localized with PCNA foci (Figure 4A). This suggests that the foci produced in the *K63R-Ub*–expressing cells are typical of those previously reported to occur at sites of blocked replication [44]. To rule out the possibility that UV differentially affects the cell cycle in the two cell lines (and thus the number of cells in the S phase), we measured cell-cycle distributions before and after UV treatment and found no significant differences (Figure S3). In both cell lines, the percentage of cells with foci after UV treatment increased rapidly during the first 30 min and then reached a plateau after 3–4 h (Figure 4B). Thus, although the percentage of positive cells was consistently higher in cells expressing *K63R-Ub,* the kinetics of foci formation are similar. This supports the argument that the K63R-Ub mutant is not interfering in some way with TLS polymerase recruitment dynamics. Interestingly, the magnitude of the increase in foci formation in the *K63R-Ub* mutant cells is similar to the increase in UV-induced mutation frequency in these cells (Figures 3B and 4B).

We also looked for possible changes in the types of mutations induced by UV irradiation after inhibition of K63-polyubiquitination. The two predominant UV-induced lesions are the *cis*-syn cyclobutane pyrimidine dimer (CPD) and the pyrimidine-6/4-pyrimidone (6–4PP) photoproduct [45,46]. The most common lesion is the thymine–thymine CPD (represented by T–T) followed by T–C and the thymine–cytosine 6–4PP (represented by T(6,4)C) [47]. Levels of T(6,4)T, C–T, and C–C lesions are comparatively much lower. However, the normal spectrum of UV-induced mutations does not match this pattern of damage induction. Mutations are primarily C to T transitions that arise at T–C and C–C sites due to mis-incorporation of adenine opposite the 3′C [48,49]. The weak contribution of the T–T lesion to mutation may be explained by the activities of POLη and POLι, which accurately bypass T–T and T(6,4)T lesions, respectively [50,51].

To further probe for possible changes in the function of these polymerases upon inhibition of K63-polyubiquitination, we examined the spectrum of UV-induced mutations in cells expressing *K63R-Ub.* By sequence analysis of the expressed *HPRT* transcript, we found that the increase in mutations noted in Figure 3 can be accounted for entirely by additional point mutations. We sequenced 20 of these mutations and found that they were all located at dipyrimidine sites (Table 1). The majority of mutations were C to T transitions (80%), with most of these being TC to TT (55%) (Figure 4C). These data are consistent with the mutation spectrum of other normal cell lines and contrast with data reported for cells with disruptions in TLS polymerases [48–50,52]. Importantly, inhibition of K63-polyubiquitination did not cause any mutations at T–T sites, suggesting normal function of both POLη and POLι in these cells.

**Table 1:**
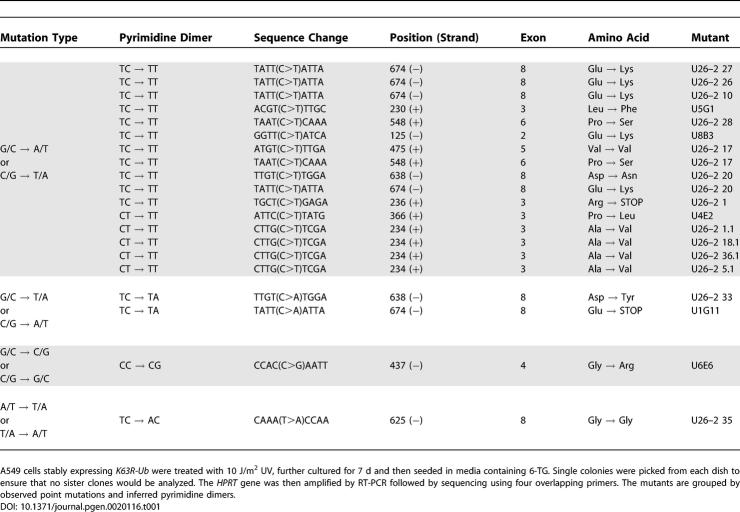
Disrupting K63-PolyUb Chain Assembly Induces a Characteristic UV Mutation Signature

Collectively, these data suggest that inhibition of K63-polyUb chain assembly results in an increased requirement for TLS after UV irradiation and consequently increased numbers of visible TLS foci, and an increase in TLS-associated mutations. To further support this assertion, we examined the dependence of the observed phenotype on RAD18 function. We transfected our *WT-Ub* and *K63R-Ub*–expressing stable cell lines with siRNA directed against RAD18 using conditions which consistently showed >10-fold reduction in expression (Figure S1). In both cell lines, UV-induced POLη foci formation was abrogated by RAD18 knockdown, implying that the recruitment of TLS polymerases to sites of damage are RAD18-dependent (Figure 4D). This is similar to previous reports showing the requirement of RAD18 for POLη foci formation [11]. Significantly, the UV-induced foci formation in *K63R-Ub*–expressing cells was also reduced to nonirradiated levels, suggesting that the increased number of foci that are found in cells expressing the *K63R* mutant is also downstream of RAD18. RAD18 has been previously shown to be important for recombinational repair, and RAD18-knockout mouse embryonic stem cells exhibit more sister chromatid exchanges in response to DNA damage [53]. The combination of disrupting K63R-polyUb chain formation and RAD18 knockdown did not show an increase in mutations; in fact a modest, but non-significant, decrease was observed (Figure 4E). The lack of an additive mutation effect supports the foci data implicating a role for K63-polyUb chain formation downstream of RAD18.

### PCNA Is Polyubiquitinated

**Figure 5:**
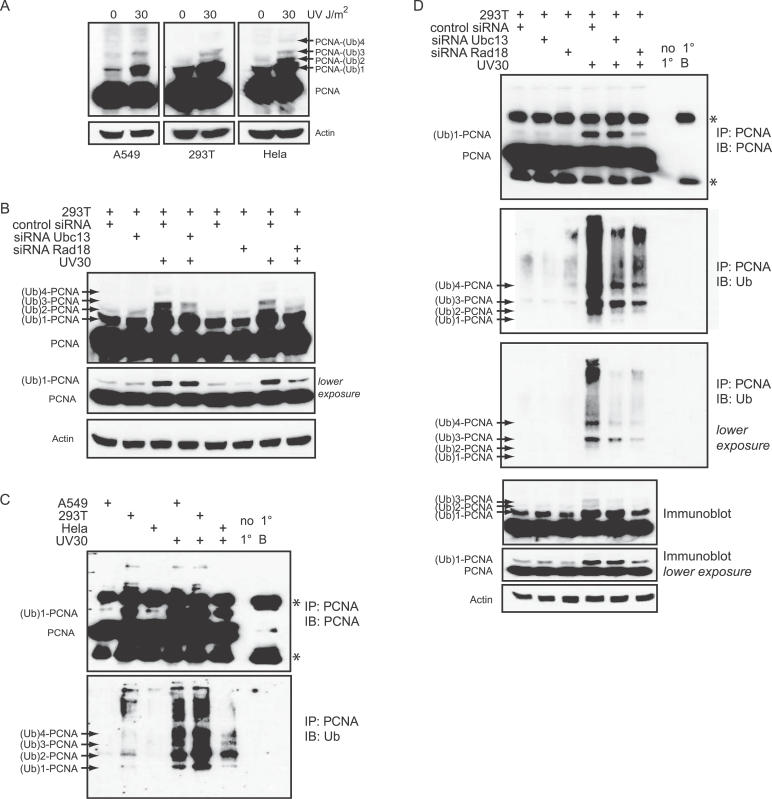
Modification of PCNA by Polyubiquitin in Human Cells after DNA Damage (A) A549, 293T, and Hela cells were irradiated with 0 or 30 J/m^2^ UV and lysed 6 h posttreatment followed by immunoblotting for PCNA. (B) 293T cells were transfected with 100 nM of either control siRNA, siRNA Ubc13, or siRNA RAD18. Seventy-two hours post-transfection, cells were treated as described in Figure 1A. A darker and lighter exposure of the PCNA immunoblot is shown. (C) A549, 293T, and Hela cells were irradiated with 30 J/m^2^ UV and lysed in boiling SDS, diluted in lysis buffer and subjected to immunoprecipitation with a PCNA antibody and detected with PCNA or Ub antibodies. The controls in the immunoprecipitations were “no 1”, in which lysates were incubated with beads but no PCNA antibody, and “1 B” in which PCNA antibody was incubated with beads alone. (D) 293T cells were transfected as described in Figure 5B. Seventy-two hours post-transfection, cells were irradiated with 30 J/m^2^ of UV and lysed 6 h later in boiling SDS, diluted in lysis buffer, and subjected to immunoprecipitation with a PCNA antibody and immunoblotted for PCNA (upper panel) and Ub (lower panel). A lighter exposure of the PCNA IP immunoblotted for Ub is also shown. A PCNA immunoblot with darker and lighter exposure performed on protein lysates from the same samples used in the immunoprecipitations is also shown. Asterisks denote immunoglobulin heavy and light chains as detected on the immunoprecipitations.

Our data support a role for the formation of K63-polyUb chains in promoting the recovery of human cells from DNA damage through an error-free pathway that is distinct from TLS. A likely target of this polyubiquitination is PCNA, which in yeast is modified by K63-polyUb via the RAD5/MMS2 complex. However, similar modification of PCNA has not been observed in UV-irradiated human cells [29]. We investigated PCNA modification after UV irradiation in three separate human cell lines: A549 lung cancer cells; 293T embryonic kidney cells; and Hela cervical cancer cells. Six hours following a dose of 30 J/m^2^, we observed the appearance of a prominent band consistent with mono-ubiquitinated PCNA, and overexposure of this blot revealed additional PCNA-immunoreactive bands of higher molecular weight consistent with PCNA modified with 2, 3, and 4 Ub molecules (Figure 5).

As it has been previously demonstrated that RAD18 is required for the mono-ubiquitination of PCNA in human cells, and that this monoUb PCNA species is required as a substrate for UBC13-mediated K63-polyubiquitination in yeast, we sought to determine whether the observed higher molecular weight bands are dependent on either RAD18 or UBC13. To this end, the expression of RAD18 or UBC13 was knocked down using the appropriate siRNAs (Figure S1). As expected, the band corresponding with monoUb PCNA was substantially reduced in lysates from RAD18 siRNA-transfected cells, and this also resulted in suppression of the higher molecular weight (polyUb) forms of PCNA presumably modified with 2, 3, or 4 Ub molecules (Figures 5B and S4A). In contrast, knockdown of the E2 Ub ligase responsible for K63-polyubiquitination had no effect on the formation of monoUb PCNA after UV irradiation, but did effectively reduce the di, tri, and quad polyUb PCNA bands to levels similar to those in the RAD18 knockdowns (Figures 5B and S4A). Together these data suggest that in both cell lines tested, UV induces modification of PCNA by both monoUb (in a RAD18-dependent manner) and by K63-polyUb chains of length 2, 3, and 4 (in a RAD18 and UBC13-dependent manner).

To further demonstrate that these higher molecular weight species are indeed ubiquitinated forms of PCNA, we immunoprecipitated PCNA from A549, 293T, and Hela cells, and probed using an antibody directed against Ub or PCNA (Figure 5C). In addition, we excluded the possibility that an ubiquitinated protein was co-immunoprecipitated with PCNA by lysing cells in boiling 0.5% sodium dodecyl sulfate (SDS) to ensure dissociation of PCNA complexes. Following a 5-fold dilution (0.1% SDS), the lysates were immunoprecipitated. Under these conditions, we reproducibly observed several higher molecular weight bands consistent with polyubiquitination of PCNA in each of the three cell lines (Figure 5C and 5D, and Figure S4B and S4C). These Ub-immunoreactive bands correspond well with the predicted molecular weights for di-, tri-, and quad-ubiquitinated PCNA. The antibody against Ub reproducibly demonstrated less affinity for the mono-ubiquitinated form of PCNA, although this was clearly the most abundant form as shown by PCNA immunoblots (Figure 5C and 5D, and Figure S4B and S4C). This appears to be a characteristic of the antibody, as we have seen this reproducibly for other ubiquitinated proteins (unpublished data).

Interestingly, each of the cell lines, particularly 293T cells, also show low levels of PCNA polyubiquitination in the absence of UV irradiation. However, in all cases the Ub-immunoreactive bands are significantly increased upon irradiation in a manner consistent with the increase in mono-ubiquitinated PCNA (Figure 5C and 5D, and Figure S4B and S4C). Similar to previous reports [29], mono-ubiquitinated PCNA was readily visible 1.5 h after UV treatment and remained present for up to 24 h as detected by the PCNA antibody (Figure S4B). Similarly, bands consistent with di, tri, and quad polyUb forms of PCNA became visible within 1.5 h following UV irradiation, and remained present up to 24 h after exposure (Figure S4B). Importantly, consistent with the PCNA Western blots (Figures 5B and S4), the Ub-immunoreactive bands following PCNA IP in both Hela (Figure S4C) and 293T (Figure 5D) cells were substantially reduced following knockdown of either RAD18 or UBC13. As expected, RAD18 knockdown blocked both mono-ubiquitination and polyubiquitination of PCNA, whereas UBC13 knockdown inhibited only the di, tri, and quad polyUb forms (Figure 5B and 5D, and Figure S4A and S4C).

Collectively, these data show that PCNA is indeed modified by polyUb chains in human cell lines. Similar modification was observed in primary skin and lung fibroblasts (unpublished data) and in response to other forms of damage such as cisplatin (Figure S5). We speculate that the lack of PCNA polyubiquitination reported earlier [29] may be explained by technical difficulties in detecting Ub owing to its strong tertiary structure [54], the low abundance of polyubiquitinated PCNA, or perhaps differences in cell types. In our studies, Ub blots were autoclaved to overcome detection problems associated with its strong tertiary structure [54]. We also excluded the possibility that DUB enzymes in cell lysates may have activity against ubiquitinated PCNA by repeating the immunoprecipitation in the presence of *N*-ethylmaleimide (NEM), a nonspecific inhibitor of DUBs (Figure S6). Under these conditions, no change in PCNA polyubiquitination was observed.

## Discussion

The highly conserved Ub protein serves as a pleiotropic covalent tag for many cellular proteins. It has essential proteolytic and nonproteolytic functions that are based on the length and topology of the chain formed. The pathway in which Ub is most commonly associated is the proteasome pathway, a system for targeting protein substrates via K48-linked polyUb chains for degradation in the 26S proteasome [55]. However, there is increasing evidence that Ub plays an important role in a number of nonproteolytic pathways including receptor internalization [56], translation [57], signal transduction [28], gene regulation [58], and DNA repair [5,6,14,25,29,59]. These roles appear to be mediated in part by the non-canonical polyUb chains. Much less is known about this aspect of ubiquitination compared with the role of K48-polyUb in protein degradation. Of particular interest are chains linked through K63, as genetic studies in S. cerevisiae have shown that the enzymatic complex (RAD5, UBC13/MMS2) that assembles these chains is required to protect cells from the harmful effects of genotoxic agents by allowing the replication machinery to bypass DNA lesions in a faithful manner [14]. In fact, ubiquitination of the DNA polymerase processivity factor PCNA is emerging as a key “molecular switch” for DDT [5,6,29]. Mono-ubiquitination of PCNA promotes error-prone TLS, while K63-polyUb activates error-free damage avoidance. The body of evidence supporting the requirement of PCNA post-translational modifications for DDT in mammalian cells is only now emerging.

**Figure 6:**
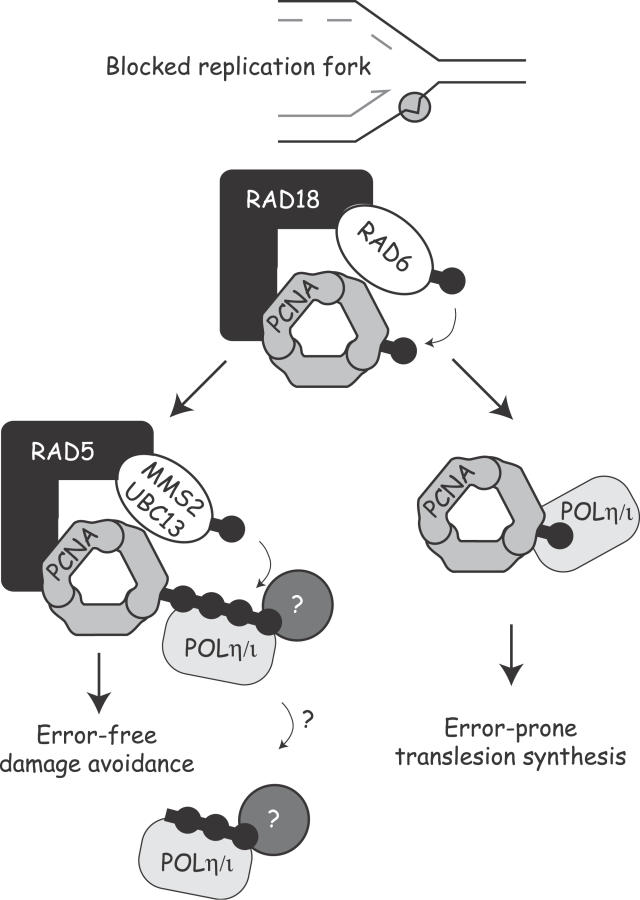
Model of the DDT Pathway in Mammalian Cells Recovery from a stalled replication fork at sites of DNA damage can occur by one of two alternative pathways. Previous work has shown that PCNA mono-ubiquitination by the RAD6/RAD18 complex stimulates lesion bypass through recruitment of the error-prone TLS polymerases. Here we show that an alternative error-free pathway requires formation of K63-polyUb chains. Blockade of this error-free pathway results in increased use of the TLS polymerases after DNA damage and a corresponding increase in mutations. As the TLS polymerases POLη and POLι both bind directly and avidly to polyUb chains [20], it is hypothesized that the interaction with K63-polyUb causes a disengagement of the polymerase from the DNA, allowing other proteins to migrate to the site of damage to perform error-free repair. This model predicts that K63-polyubiquitination acts to suppress environmental carcinogenesis by preventing genomic instability that would otherwise be introduced by the TLS polymerases.

In this report, we provide evidence to support a model (Figure 6) in which ubiquitination of PCNA acts at a central decision point to direct the recovery of blocked replication forks towards one of two alternative pathways in mammalian cells. Recent reports have confirmed that RAD6-dependent mono-ubiquitination of PCNA also stimulates TLS in human cells. This stimulation appears to result through direct binding of the TLS polymerases to mono-ubiquitinated PCNA [11,29].

Our data indicate that formation of K63-polyUb chains is required to utilize an error-free pathway distinct from TLS. This pathway is required for cell survival from at least some types of DNA damage, as its inhibition cannot be compensated for by the alternative TLS pathway in the case of cisplatin-damaged cells. For UV-induced damage, inhibition of K63-polyubiquitination does not affect overall cell survival, but instead causes an increase in mutations arising from an apparent increased requirement for the error-prone branch of TLS. This is supported by several lines of evidence. First, blockade of K63-polyUb chain formation led to a 2.4-fold increase in RAD18-dependent TLS foci after UV irradiation. Second, we found that the number of UV-induced mutations increased by a similar factor in these cells, and that the spectra of these mutations are consistent with that produced normally by error-prone TLS polymerases after UV treatment. Third, POLη knockdown in combination with blockade of K63-polyUb chain formation led to increased toxicity to UV irradiation, although no change was seen with either individually. Fourth, an increased reliance on the TLS arm upon blockade of K63-polyUb chain assembly was revealed by a synergistic increase in UV-induced mutations when expressed in POLη knockdown cells. POLη knockdown cells showed a high mutation rate as expected, but this rate increased by a factor of 3.5 when K63-polyUb chain assembly was inhibited.

Together, these data imply that formation of K63-polyUb chains can activate an error-free mechanism to protect cells against mutations that would otherwise be induced by the error-prone TLS polymerases. It will be of interest to determine whether K63-polyUb chain formation also plays a role in protection against sunlight-induced skin cancer.

An obvious question that emerges is how formation of K63-polyUb acts to suppress TLS. Recent reports have demonstrated that the TLS polymerases POLη and POLι both bind directly and avidly to polyUb chains through newly discovered binding domains [20,60]. A C-terminal zinc finger domain of POLη and the proline residue at position 692 of POLι are required for the respective interaction with Ub [20]. Together with our data, this suggests a possible mechanism whereby differential ubiquitination of PCNA could act as a switch between TLS and an alternative error-free pathway (Figure 6). In this model, the TLS polymerases are recruited to the sites of replication through interaction with mono-ubiquitinated PCNA and subsequently mediate TLS across DNA lesions. Extension of the Ub chain through K63-linked polyubiquitination in some way suppresses TLS activity and promotes recovery through an alternative error-free pathway. This suppression may be mediated through the recently discovered ability of POLη and POLι to directly bind K63-polyUb chains. An intriguing possibility is that K63-polyUb chains are cleaved upon binding to TLS polymerases, thereby functionally removing them from the site of the lesion. This possibility is supported by the low detectable levels of polyubiquitinated PCNA as well as by the observed increase in POLη foci in *K63R-Ub*–expressing cells.

Although our data suggest that PCNA is indeed a target for K63-polyubiquitination, they do not exclude the possibility that other key proteins in this pathway are also important substrates for these chains. Indeed, K63-polyubiquitination occurs on at least three proteins (RIP, NEMO, and TRAF6) in an unrelated pathway that activates NF-κB [28,61,62]. In this pathway, K63-polyUb chains on multiple proteins may facilitate their assembly into an active complex [62]. It is therefore intriguing to speculate that K63-polyUb chains may not only uncouple the TLS polymerases from the site of damage, but may also provide a mechanism for recruitment of other proteins required for error-free repair.

Non-proteolytic roles for Ub have also been implicated in other DNA-repair pathways that may interact with DDT, most notably that involving Fanconi's anemia (FA) gene products [18]. FANCD2 becomes mono-ubiquitinated after DNA damage and localizes to nuclear foci [63]. FANCC has been associated with the TLS polymerases REV1 and REV3 [64] and may also interact with the BLM helicase [65], a candidate for promoting fork reversal in the error-free damage-avoidance pathway [66]. A challenge for future investigations will be to understand how K63-polyUb chain assembly is regulated and how these chains promote interaction with other pathways to mediate error-free recovery from DNA damage.

## Materials and Methods

### Cell culture and treatments.

The construction of the Ub-expressing plasmids has been described elsewhere [31]. The *POLη-GFP* plasmid was a gift of Dr. Alan R. Lehmann, (Genome Damage and Stability Centre, University of Sussex, Falmer, Brighton, United Kingdom). All cell lines were cultured in DMEM (Sigma, St. Louis, Missouri, United States) supplemented with 10% FBS (Sigma). A549 cells were co-transfected with *WT-Ub-GFP* or *K63R-Ub-GFP* plasmids and the *pBabePuro* plasmid (for selection) using FuGene 6 (Roche, Basel, Switzerland). HeLa cells were transfected with *WT-Ub-puro* or *K63R-Ub-puro* constructs using lipofectamine (Invitrogen, Carlsbad, California, United States). Stable transfectants were selected in 1 μg/ml puromycin (Sigma) and/or by flow cytometry (FACSAria, BD Biosciences Pharmingen, San Diego, California, United States).

The sensitivity to UV irradiation alone, UV combined with caffeine, and cisplatin alone was evaluated using clonogenic survival assays. UV irradiation was performed on 80% confluent cells in 6-cm dishes using a UVC (254-nm) germicidal lamp at a dose rate of 1 J/m^2^/s. UV and caffeine combination studies were carried out as above, but cells were plated in 0.4 mM caffeine immediately after UV irradiation. Cells were treated for 1 h in cisplatin diluted in culture media. Cells were plated in 6-cm dishes in triplicate and incubated for 2 wk to obtain colony formation. Colonies were fixed, stained with 2% bromophenol blue in 70% ethanol, and colonies containing ≥50 cells were counted. All experiments were normalized for plating efficiency.

The sensitivity to UV irradiation in POLη knockdown cells was performed as above with the exception that cells were transfected twice with SiGenome Smartpool reagent specific for human POLη (Dharmacon Research, Lafayette, Colorada, United States) using oligofectamine (Invitrogen). The transfections were carried out 72 and 24 h before UV treatment to achieve optimal long-term knockdown as determined by quantitative RT-PCR.

Quantitation of gene expression was performed using an Applied Biosystems (Foster City, California, United States) 7500 Real-Time PCR system using their “assay on demand” technology. *RAD18* expression was determined with the Hs00220119_m1 probe, *POLη* with the Hs00197814 probe, and *18S* with the Hs99999901_s1 probe. Reactions were performed using Taqman Universal PCR Master Mix from Applied Biosystems.

### Immunoblotting.

Following the indicated treatments with either UV irradiation, cisplatin, and/or SiGenome Smartpool reagent specific for human UBC13 or human RAD18 (Dharmacon), cells were harvested in lysis buffer (20 mM Tris-HCl [pH 7.5], 150 mM NaCl, 1% Triton-X-100, 2 mM EDTA, and 5% glycerol with 200 μg/ml phenylmethylsulfonyl fluoride, 2 mM NaVO_4_, 2 mM NaF, and 2 mM NaPPi protease-inhibitor cocktail). Samples were sonicated, soluble fractions were recovered, and proteins were quantified using the Bradford protein assay (Bio-Rad). Proteins were resolved on either a single-phase (10%) or two-phase SDS-polyacrylamide gel (10% and 15%) and electroblotted onto a Hybond C nitrocellulose membrane (Amersham Pharmacia Biotech, Piscataway, New Jersey, United States). The membrane was stained with Ponceau S (Sigma) prior to Western blotting with the indicated primary antibody. The following antibodies were used: rabbit polyclonal Ub (Dako, Glostrup, Denmark), mouse monoclonal RGS-His (Qiagen, Valencia, California, United States), mouse monoclonal PCNA PC10 (Chemicon, http://www.chemicon.com), rabbit polyclonal GFP (Santa Cruz Biotechnology, Santa Cruz, California, United States), and mouse monoclonal actin (Sigma). Proteins were visualized by a horseradish peroxidase method using ECL (Kirkegaard and Perry Laboratories, http://www.kpl.com).

### Immunoprecipitation.

Cells were UV-irradiated with 30 J/m^2^ as described above and either left untreated or transfected with SiGenome Smartpool reagent specific for human UBC13 or human RAD18 (Dharmacon). Cells were lysed (6 h after irradiation) in lysis buffer supplemented with 0.5% SDS. Lysates were sonicated and boiled for 5 min followed by dilution to 0.1% SDS. After protein quantitation, 500 μg of protein was incubated overnight at 4 °C with anti-PCNA (1/200). The following day, lysates were incubated for 48 h at 4 °C with 100 μl of Gamma-Bound Sepharose Beads (Amersham Pharmacia Biotech). Beads were washed extensively in lysis buffer, and proteins were eluted by boiling in Laemmli's SDS sample buffer. Immunoblotting was performed as described above except that the membranes were autoclaved for 20 min in ddH_2_O after protein transfer, and proteins were visualized using SuperSignal West Pico Chemiluminescent Substrate (Pierce Biotechnology, Rockford, Illinois, United States).

### Mutation spectrum.

To eliminate background *HPRT* mutations, cells were cultured in hypoxanthine, aminopterin, and thymidine (HAT)–supplemented culture medium for 1 wk. UV-induced *HPRT* mutants were obtained by seeding 1.5 × 10^4^ cells in 24-well plates, followed by 10 J/m^2^ UV irradiation 24 h later. Cells were subcultured for 7 d, and re-seeded at 5.0 × 10^4^ cells on 35-mm dishes in medium containing 30 μM 6-thioguanine (6-TG). Individual colonies were picked and grown until enough cells were obtained for RNA isolation using RNA-aqueous kit (Ambion, Austin, Texas, United States). The *HPRT* gene was subjected to RT-PCR, followed by sequencing using the following overlapping primers: HPRT1–5′CTTCCTCCTCCTGAGCAGTC3′; HPRT2–5′AAGCAGATGGCCACAGAACT3′; HPRT3–5′CCTGGCGTCGTGATTAGTG3′; HPRT4–5′TTTACTGGCGATGTCAATAGGA3′; HPRT5–5′GACCAGTCAACAGGGGACAT3′; and HPRT6 5′ATGTCCCCTGTTGACTGGTC3′.

### Mutation frequency.

*HPRT* mutant–free cells (1.0 × 10^6^) were seeded and treated the following day with either UV irradiation (0, 5, and 10 J/m^2^) or cisplatin (0, 5, and 10 μM for 1 h). After subculturing the treated cells for 1 wk, 4.0 × 10^5^ cells were seeded in selective medium containing 6-TG (as above) and incubated until colonies were formed. Colonies were counted and *HPRT* mutation frequency was defined after correcting for plating efficiency.

Mutation frequency in response to UV treatment in POLη and RAD18 knockdown cells was performed as above with the exception that cells were transfected twice with SiGenome Smartpool reagent specific for human POLη or human RAD18 (Dharmacon) using oligofectamine. The transfections were performed 72 and 24 h before UV treatment to achieve optimal long-term knockdown as determined by quantitative PCR.

### Foci.

A549 and Hela cells stably expressing *WT-Ub-puro* and *K63R-Ub-puro* were transiently transfected with a *POLη-GFP* plasmid. Twenty-four hours post-transfection, cells were UV-irradiated at a dose of 10 J/m^2^. To observe living cells, cells were cultured in 35-mm glass-bottomed dishes (MatTek, http://www.mattek.com) with coverslips. Real-time excitation measurements to monitor fluorescent signals in transfected cells were subsequently performed using a live-cell microscopy unit mounted on a Leica DR IRBE inverted microscope (Wetzlar, Germany), equipped with a polychromator that allows generation of light of the required wavelength, using a 63× objective. Both the polychromator and filterwheel were controlled via the PC using specialized Openlab software from Improvision (http://www.improvision.com/products/openlab). At least 100 cells were counted for each cell line at each time point per experiment by a blinded independent observer.

The recruitment of POLη to foci was determined in response to UV irradiation in RAD18 knockdown cells performed as above with the exception that cells were transfected twice with SiGenome Smartpool reagent specific for human RAD18 using oligofectamine. The transfections were performed 72 and 24 h before UV treatment to achieve optimal long-term knockdown as determined by quantitative PCR.

For colocalization studies, Hela cells stably expressing *WT-Ub-puro* and *K63R-Ub-puro* were transiently transfected with a *POLη-GFP* plasmid in a chamber slide (BD Biosciences Pharmingen). Twenty-four hours post-transfection, cells were UV-irradiated at a dose of 10 J/m^2^. For detection of PCNA and POLη, cells were fixed in cold methanol for 20 min at −20 °C followed by 30 sec in cold acetone. Cells were washed twice with PBS and then incubated at room temperature with both anti-PCNA and anti-POLη. After 1 h, cells were washed with PBS and then incubated with FITC-conjugated goat antimouse IgG (Invitrogen) and Texas red–conjugated goat antirabbit (Invitrogen), for 45 min. After washing in PBS, cells were dehydrated for 1 min in 70% ethanol followed by two 1-min incubations in 100% ethanol. Cells were then mounted with Fluorescent Mounting Media (Dako) and visualized by confocal microscopy.

## Supporting Information

Figure S1 Knockdown of UBC13, RAD18, and POLη (A) Hela cells were transfected with siRNA against UBC13 and analyzed by Western blot.
(B) A549 cells were transfected twice (48 h apart) with siRNA against RAD18 or POLη. Knockdown was analyzed 24 and 72 h post–second transfection for mRNA expression relative to 18S rRNA using quantitative RT-PCR.
(28 KB PDF)

Figure S2 Increase in POLη Foci Is Also Observed in A549 Cells A549 cells stably expressing *WT-Ub* or *K63R-Ub* were treated as described in Figure 4. Two independent experiments were performed.
(16 KB PDF)

Figure S3 Cell-Cycle Profile following UV Treatment (A) A549 cells expressing *WT-Ub-GFP* or *K63R-Ub-GFP* were treated with the indicated dose of UV irradiation.
(B) Hela cells expressing *WT-Ub-puro* or *K63R-Ub-puro* were treated with 10 J/m^2^ UV irradiation and fixed either immediately or 6 h posttreatment. Following propidium iodide staining, cells were analyzed for DNA content using a FACSAria flow cytometer (BD Biosciences Pharmingen).
(33 KB PDF)

Figure S4 Modification of PCNA by Polyubiquitin in Human Cells after DNA Damage (A) Hela cells were subjected to the same procedure as carrried out in Figure 5B**.** A darker (upper panel) and lighter exposure (lower panel) of the PCNA immunoblot is shown.
(B) A549 cells were UV-irradiated as described in Figure 5A and lysed at the indicated times posttreatment. Whole-cell lysates were subjected to immunoprecipitation with an anti-PCNA antibody followed by immunoblotting for PCNA (upper panel) and Ub (lower panel). The controls in the immunoprecipitations are the same as carried out in Figure 5C. −UV indicates no UV treatment.
(C) Hela cells were subjected to the same procedure as performed in Figure 5D. A lighter exposure of the PCNA IP immunoblotted for Ub is shown. A PCNA immunoblot with darker and lighter exposure performed on protein lysates from the same samples used in the immunoprecipitations is also shown.
(67 KB PDF)

Figure S5 Cisplatin Treatment also Induces Modification of PCNA by Polyubiquitin in Human Cells Untreated, 30 J/m^2^ UV-irradiated, and 160 μM cisplatin-treated A549 and Hela cells were lysed 6 h posttreatment followed by immunoblotting for PCNA.
(33 KB PDF)

Figure S6 Inhibition of DUB Enzymes Does Not Affect Appearance of PolyUb-PCNA A549, 293T, and Hela cells were treated with 30 J/m^2^ UV irradiation and lysed in the presence or absence of the general thiol protease-inhibitor NEM. Immunoprecipitation and Western blots were carried out as described in Materials and Methods. The controls in the immunoprecipitations were “no 1”, in which lysates were incubated with beads but no PCNA antibody, and “1 B”, in which PCNA antibody was incubated with beads alone.
(30 KB PDF)

### Accession Numbers

The Entrez Gene (http://www.ncbi.nlm.nih.gov/entrez/query.fcgi?CMD=search&DB=gene) accession numbers for the gene and gene products discussed in this paper are BLM (641), CROC1 (7335), FANCC (2176), FANCD2 (2177), HPRT (3251), MMS2 (7336), NEMO (8517), NF-kappaB (4790), PCNA (5111), POLH (5429), POLI (11201), RAD18 (56852), RAD5 (850719), REV3 (5980), TRAF6 (7189), UBA52 (7311), UBA80 (6233), UBB (7314), UBC (7316), and UBC13 (7334).

## Abbreviations

6–4PP: pyrimidine-6/4-pyrimidone
CPD: cis-syn cyclobutane pyrimidine dimer
DDT: DNA damage tolerance
DUB: deubiquitinating
FA: Fanconi's anemia
HAT: hypoxanthine, aminopterin, and thymidine
K63-polyUb: lysine 63-linked polyubiquitin
MMS: methyl methane sulfonate
NEM: *N*-ethylmaleimide
PCNA: proliferating cell nuclear antigen
SDS: sodium dodecyl sulfate
TLS: translesion synthesis
Ub: ubiquitin
UBD: ubiquitin-binding domain
UBZ: ubiquitin-binding zinc-finger
WT: wild-type
XP: Xeroderma Pigmentosum
XPV: Xeroderma Pigmentosum variant
