# Supplementary material for: Lysine 63-Polyubiquitination Guards against Translesion Synthesis–Induced Mutations
Source: PLoS Genet. 2006 Jul 21;2(7):e116. doi: 10.1371/journal.pgen.0020116 (PMC1513265; doi:10.1371/journal.pgen.0020116)
Supplement: Supplementary file 6 [file pgen.0020116.sg006.pdf]

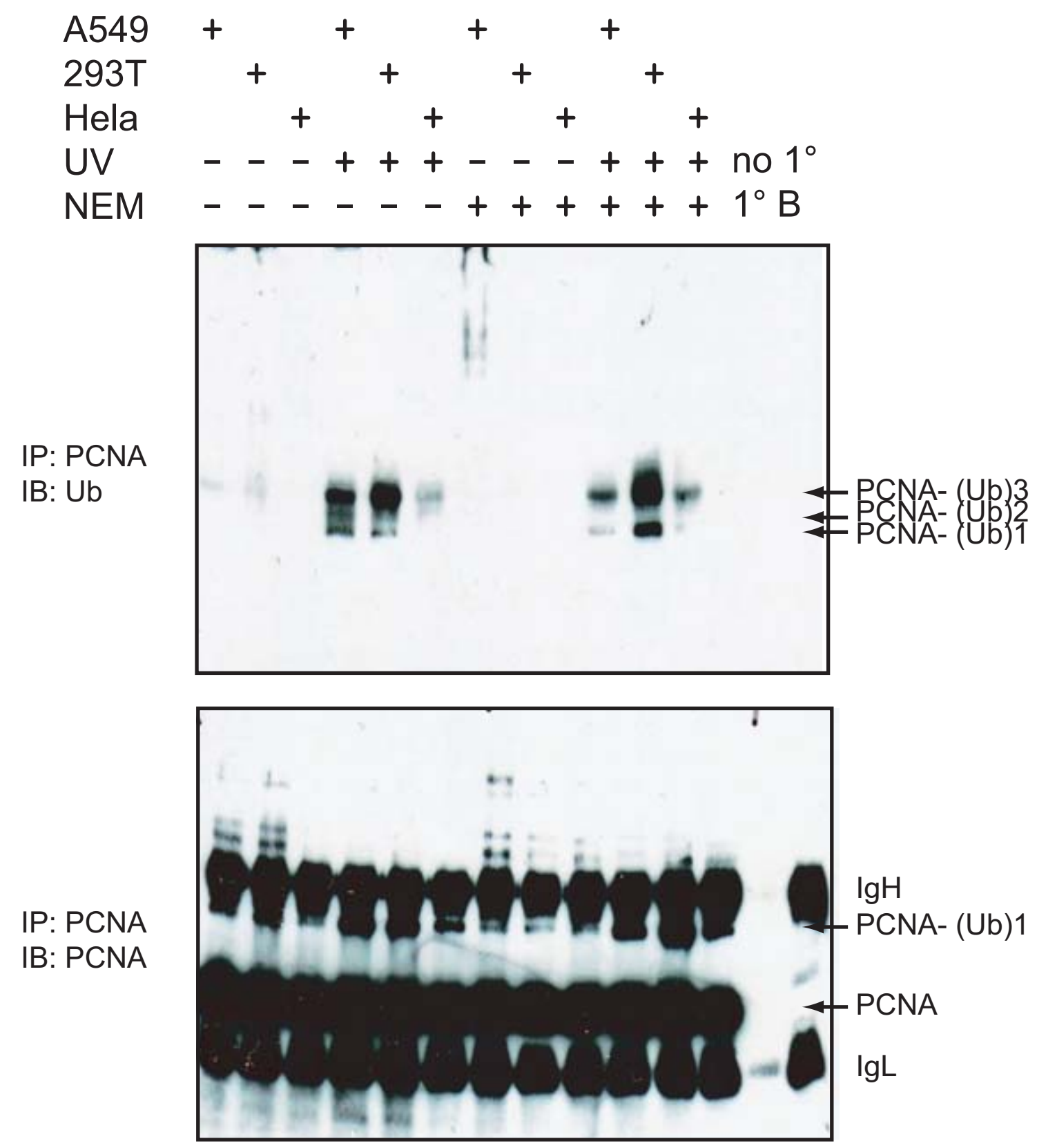

Supplementary Figure S6 *Chiu et al*
